# Chitosan-based films with cannabis oil as a base material for wound dressing application

**DOI:** 10.1038/s41598-022-23506-0

**Published:** 2022-11-04

**Authors:** Dorota Chelminiak-Dudkiewicz, Aleksander Smolarkiewicz-Wyczachowski, Kinga Mylkie, Magdalena Wujak, Dariusz T. Mlynarczyk, Pawel Nowak, Szymon Bocian, Tomasz Goslinski, Marta Ziegler-Borowska

**Affiliations:** 1 Department of Biomedical Chemistry and Polymer Science, Medicinal Chemistry Research Group, Faculty of Chemistry, Nicolaus Copernicus University in Torun, Gagarina 7, 87-100 Torun, Poland; 2 Department of Medicinal Chemistry, Faculty of Pharmacy, Nicolaus Copernicus University in Torun, Collegium Medicum, Jurasza 2, 85-089 Bydgoszcz, Poland; 3 Chair and Department of Chemical Technology of Drugs, Poznan University of Medical Sciences, Grunwaldzka 6, 60-780 Poznan, Poland; 4 Department of Environmental Chemistry and Bioanalysis, Faculty of Chemistry, Nicolaus Copernicus University in Torun, Gagarina 7, 87-100 Torun, Poland

**Keywords:** Biomedical materials, Biopolymers

## Abstract

This study focuses on obtaining and characterizing novel chitosan-based biomaterials containing cannabis oil to potentially promote wound healing. The primary active substance in cannabis oil is the non-psychoactive cannabidiol, which has many beneficial properties. In this study, three chitosan-based films containing different concentrations of cannabis oil were prepared. As the amount of oil increased, the obtained biomaterials became rougher as tested by atomic force microscopy. Such rough surfaces promote protein adsorption, confirmed by experiments assessing the interaction between human albumin with the obtained materials. Increased oil concentration also improved the films' mechanical parameters, swelling capacity, and hydrophilic properties, which were checked by the wetting angle measurement. On the other hand, higher oil content resulted in decreased water vapour permeability, which is essential in wound dressing. Furthermore, the prepared films were subjected to an acute toxicity test using a Microtox. Significantly, the film's increased cannabis oil content enhanced the antimicrobial effect against *A. fischeri* for films in direct contact with bacteria. More importantly, cell culture studies revealed that the obtained materials are biocompatible and, therefore, they might be potential candidates for application in wound dressing materials.

## Introduction

Skin damage is one of the most common injuries worldwide. In recent years, accidental skin harm has increased rapidly, leading to many patients with cutaneous lesion disease. *Skin wound healing* is a complex biological process divided into four overlapping phases: hemostasis, inflammation, proliferation, and remodeling^1^. Appropriate healing materials are necessary to promote biological healing processes by allowing gas exchange, reducing inflammation, and preventing infection. In addition, these materials should be non-toxic, have good mechanical properties, and have a satisfactory water vapour transmission rate^2^. Although many materials have been developed to accelerate wound healing, obtaining a single dressing that meets all these conditions remain challenging. Currently, many dressings consist mainly of synthetic polymers such as polyethylene glycol, poly(glycolic acid) (PGA), poly(lactide-co-glycolide), polycaprolactone (PCL), and polyurethane^3–7^. However, these materials still display various limitations that can potentially lead to infection or damage to the skin. For example, the main drawback of PCL and PGA is their hydrophobic nature and inability to encourage cell adhesion and proliferation^8^. In addition, PCL exhibits poor antimicrobial properties^9^. Natural polymers that show compatibility with the human body and biological processes seem to be a better solution.

Some biopolymers such as chitosan, cellulose, collagen, alginate, and keratin have been studied as dressing materials, e.g., films, foams, and hydrogels^10–12^. However, like synthetic polymers, most natural polymers do not satisfy the essential prerequisites of a good wound dressing. For example, studies show that alginate dressings are ineffective hemostatic materials, especially in cases of massive bleeding^13^. Another example is bacterial cellulose, which exhibits terrible inherent antibacterial activity, and primarily limits its use as a dressing material^14^. Chitosan is considered a preferred material due to its high molecular weight, excellent biocompatibility, and hemostatic properties, making it widely used in wound healing. This biopolymer is obtained from chitin by its alkaline deacetylation^15^. Also, chitosan is an inexpensive polymer making it affordable for patients who frequently use wound dressings. Furthermore, the properties of chitosan make it possible to form so-called active dressings that control the biochemical state of the wound. However, the risk of infection is still present. To prevent this, it is necessary to incorporate active substances into such materials further to improve the properties of bioactive wound dressings. In recent years, attention has been drawn to the antimicrobial activity of antibiotics and chemotherapeutic agents in wound healing^16–19^. However, these compounds are not entirely safe as they may cause various side effects, and their excessive use can lead to microbial resistance. According to the reports published by the World Health Organization (WHO), the number of people struggling with antibiotic resistance remains extremely high worldwide^20^. Hence, an alternative approach is required. Many natural active substances reveal antibacterial properties and might have potential use in treating wound infections. Recent studies have reported using various natural agents for wound healing, such as honey, aloe vera, quercetin, thymol, and curcumin^21–24^. However, the application of cannabidiol is also an area worth exploring.

Bioactive cannabidiol (CBD) (see Supplementary Fig. S1 online) is a cannabinoid derived from the *Cannabis sativa* plant, and unlike tetrahydrocannabinol (THC), it does not show any psychoactive effects. CBD has attracted widespread scientific and industrial interest because it exerts many therapeutic effects, including anticonvulsant, antipsychotic, anticancer, anti-inflammatory, and neuroprotective activities^25–28^. Despite CBD's broad spectrum of effects, there are currently only a few licensed medicinal products available, including one approved product in the United States based on highly purified cannabidiol to treat Lennox-Gastaut and Dravet syndromes^29^. However, studies to date have focused on the oral administration of cannabidiol (including in combination with psychoactive THC) to treat different sources of pain^30,31^. CBD's properties (such as antioxidant, antibacterial, anti-inflammatory, regenerative, and blood-clotting properties)^32–34^ make it a promising agent for promoting wound healing. Moreover, cannabidiol is lipophilic and is easily absorbed into all types of cutaneous wounds, and of equal importance is the fact that CBD can reduce the production of reactive oxygen species^35^, which is essential to promote wound healing.

Cannabidiol can come in various formulations (capsules, chewing gums, sprays). However, the most interesting product seems to be cannabis oil, which contains CBD mixed with a base (carrier) oil such as coconut oil or cannabis seed oil. CBD oil also contains a wide variety of fatty acids, proteins, amino acids, vitamins A, C, and E, β-carotene, and minerals, specifically phosphorus, potassium, magnesium, sulfur, and calcium^36^. Owing to these, CBD oil can be used as a remedy for skin lesions. Moreover, unsaturated fatty acids accelerate wound healing processes and reduce inflammation^37,38^. Cannabis oil is also readily available, which is an additional advantage.

In this study, novel chitosan-based biomaterials containing cannabidiol were obtained, with cannabis oil being used as a source of cannabidiol. A complete characterization of the prepared films, including surface morphology, chemical structure, thermal stability, mechanical properties, and CBD release, was carried out. The extent of protein adsorption (human serum albumin) on the surface of the obtained biomaterials was evaluated. Furthermore, the preliminary acute toxicity of the prepared samples was studied using the Microtox test. The results suggest that the cannabidiol-containing chitosan-based films (CBD-CS) possess the most prerequisites for a good dressing for wound healing applications.

## Experimental

### Materials

Chitosan (the low molecular weight, deacetylation degree of 79%), phosphate-buffered saline (PBS; KH_2_PO_4_, K_2_HPO_4_) (pH 7.4), human serum albumin (HSA), 2,2-diphenyl-1-picrylhydrazyl radical (DPPH), reagents for Winkler method, glycerin and diiodomethane (pure for analysis), methanol gradient grade were purchased from Sigma-Aldrich (Munich, Germany). Water was purified with a Milli-Q Water Purification System (Millipore Corp., Bedford, MA). 20% Cannabis oil (CBD Pure) was purchased from a local pharmacy.

All reagents for the fibroblast cell culture: Eagle's Minimum Essential Medium (EMEM), fetal bovine serum (FBS), Non-Essential Amino Acids (NEAA), L-glutamine, trypsin–EDTA, penicillin/streptomycin solution, Dulbecco’s phosphate buffer saline (DPBS) were purchased from Biowest (Nuaillé, France). 2-Propanol and hydrochloric acid (pure for analysis) and MTT (3-(4,5-dimethylthiazol-2-yl)-2,5-diphenyltetrazolium bromide; suitable for cell culture), were purchased from Sigma Aldrich.

### Preparation of cannabidiol-chitosan-based films (CBD-CS)

Chitosan (1.0 g) was stirred in acetic acid solution (C = 1%, 100 mL) at room temperature until it dissolved. After a clear solution was formed, 0.01 g of cannabidiol oil (1% by weight to chitosan) was added, and the stirring continued for 30 min. Next, the solution was poured into a polystyrene Petri dish (55 mm diameter) and left to the solvent to evaporate. The residue was left at room temperature to dry. The obtained CBD-CS film was removed from the dish and marked as 1CBD-CS.

In the same way, chitosan films were prepared with 5 and 10% by weight CBD by adding to the chitosan solution 0.05 and 0.1 g of CBD oil, respectively. The obtained films were marked as 5CBD-CS and 10CBD-CS.

### Methods

#### Characterization of the CBD-CS films

The ATR-FTIR spectra of the chitosan and CBD-CS films were recorded at room temperature using the spectrophotometer Spectrum-Two (Perkin Elmer, Waltham, MA, USA) equipped with a diamond crystal in the range of 4000–450 cm^−1^, resolution 16 cm^−1^, and 64 scans.

The morphology of the obtained biomaterials was studied with a Scanning Electron Microscope (1430 VP LEO Electron Microscopy Ltd.) and Atomic Force Microscopy (AFM) (MultiMode Nanoscope IIIa Veeco Metrology Inc., USA) technique. Roughness parameters were calculated for the 5 × 5 μm^2^ scanning area.

Thermal Gravimetric Analysis of the CBD-CS films was performed on a TA Instruments (SDT 2960 Simultaneous DSC-TGA thermogravimetric analyzer) at a 10 °C/min heating rate in the range from ambient to 600 °C in the atmosphere of nitrogen.

The contact angle (ϴ) of pure chitosan and CBD-CS films was performed at 22 °C by the sessile drop method using a DSA10 goniometer (Kruss GmbH, Germany). Three types of liquid were used: water, glycerin (see Supplementary Fig. S1 online), and diiodomethane. At least three measurements were performed to assess each sample, and the surface free energy (γs) was calculated by the standard Owens–Wendt method^39^.

The mechanical properties of pure chitosan and CBD-CS films were tested at room temperature by the EZ-Test E2-LX Shimadzu texture analyzer (Shimadzu, Kyoto, Japan). Five sample strips (50 mm in length and 4.5 mm in width) of chitosan and cannabidiol-chitosan films were cut and clamped between pneumatic grips. The tests were performed at an extension rate of 20 mm/min, and each measurement was repeated three times.

For the degradation study, the pre-weight and UV light sterilized pure chitosan and CBD-CS films were immersed in PBS and incubated at 37 °C for 14 days. The samples were taken out, dried, and weighed after every day. This experiment was performed in triplicate. The following equation calculated the degradation rate:

1 $$ Degradation\,rate = \frac{{\left( {W_{0} - W} \right)}}{{W_{0} }}*100\% $$

where W_0_ is the initial weight of the film and W is the final weight of the film^40^.

The pure chitosan and CBD-chitosan films were placed in PBS for 24 h at room temperature for the swelling study. The samples were weighed immediately after removing them from the solution. This experiment was performed in triplicate. The following equation calculated the degradation rate:

2 $$ Swelling\,rate = \frac{{\left( {W_{wet} - W_{Dry} } \right)}}{{W_{Dry} }}*100\% $$

where W_wet_ is the weight of the swollen film and W_Dry_ is the weight of dried film^41^.

#### Antioxidant effects

The antioxidant activity of the CBD-CS samples was evaluated by the DPPH free radical scavenging method, as previously described^42^. First, 1.0 mM DPPH in ethanol was prepared, and then 30 μL of this solution was added to the liquid film precursor solution. After 30 min incubation at room temperature, the absorbance was recorded against a blank at 517 nm using a UV-1800 spectrophotometer (Shimadzu, Japan). The experiment was performed three times.

#### The water vapor transmission rate (WVTR)

The WVTR of the obtained materials was investigated according to the previously reported method^43^. Firstly, a desiccant was prepared. For this, calcium chloride was dried at 100 °C for 24 h. Then, the freshly prepared desiccant was poured into a plastic box. The CBD-CS samples were cut into round shapes and then placed on the top surfaces of the box. The bottles were sealed tightly. The opened bottles with desiccant were left as control samples. After 24, 48, and 72 h, the samples were removed, and the mass of the desiccant with adsorbed water was measured. WVTR was calculated using the following equation:

3 $$ WVTR = \frac{{\left( {\frac{\Delta w}{{\Delta t}}} \right)}}{A} $$

where $\left(\frac{\Delta w}{\Delta t}\right)$ denoted the slope of the plot, and A denoted the effective transfer area.

#### Oxygen permeability

The oxygen permeability of the CBD-CS films was determined by measuring the dissolved oxygen in the distilled water according to Winkler’s method^44^. Freshly boiled distilled water (200 mL) was added to the bottle, and the films were fixed on top of the bottle. The positive control was an open bottle, while the negative control was a closed bottle with an airtight cap. All samples were left in ambient conditions for 24 h. The experiment was performed three times. The results were expressed as the amount of dissolved oxygen (mg/mL).

#### Release of CBD from the obtained materials

Chromatographic analyses were performed on the Kinetex C18 column (150 × 4.6 mm) with a particle size of 5 µm using a methanol–water mobile phase in gradient elution at 30 °C. The gradient profile was as follows: initial condition 60% of MeOH in water, next the increase of MeOH up to 95% during 4 min. Finally, 95% of MeOH was maintained to the end of the analysis within 15 min. The detection wavelength was set at 254 nm. 10 µL of a sample was injected in each analysis. Quantitative analysis was performed using the calibration curve. The applied method was linear in the range from 2 µg/mL to 2.5 mg/mL with the coefficient R^2^ equal to 0.999. The chromatographic experiments were performed on the Shimadzu Prominence LC (Kyoto, Japan). The liquid chromatograph consists of a quaternary gradient pump (LC-20AD), an autosampler (SIL-20C), a column thermostat (CTO-10AS), and a spectrophotometric diode-array UV–Vis detector (SPD-M20A). Instrument control, data acquisitions, and processing were performed with LabSolutions software for HPLC.

#### Protein adsorption

Adsorption of proteins to the obtained biomaterials was determined using a fluorescence method. First, an HSA solution was prepared in 50 mM phosphate buffer pH 7.4 at a concentration of 6.13 μM. Then, the CBD-CS films (1 × 1 cm) at different mass concentrations were immersed in two milliliters of the freshly prepared HSA solution and incubated at 36 °C while shaken at 600 rpm. Fluorescence spectra were recorded at 298 K, intervals ranging from 290 to 400 nm at an excitation wavelength of 280 nm using a Jasco FP-8300 spectrofluorometer (Jasco, Tokyo, Japan). The spectrum was recorded within 285–400 nm at a scanning speed of 100 nm/min and Em/Ex bandwidth of 2.5 nm/5 nm. The measurement was repeated three times.

#### Microtox® test

The Microtox acute toxicity test was performed using Microtox M500 and Modern Water Microtox Omni 4.2 software. Two experiments were performed: (i) with the films dispersed in water and (ii) with the whole film fragments. For the experiments with the dispersed films, the film fragments (~ 60 mg) were stirred for 20 h in 100 mL of deionized water, and the resulting suspensions were subjected to the 81.9% Screening Test protocol supplied by the producer. The procedure was modified as described before in the case of the whole film fragments experiments^45^. Briefly, the *Aliivibrio fischeri* bacterial suspensions were prepared for the control experiment, but in each culture, a film fragment was immersed, and the bioluminescence measurements for the resulting mixtures were performed as in the 81.9% Screening Test protocol (after 5 and 15 min). All the experiments were performed in triplicate.

#### Biocompatibility evaluation using L929 mouse fibroblasts

L929 mouse fibroblasts (NCTC clone 929; European Collection of Authenticated Cell Cultures, Salisbury, United Kingdom) were used to evaluate the biocompatibility of the tested biomaterials. The cells were grown in the EMEM medium supplemented with 2 mM L-glutamine, 1% NEAA, 10% FBS and antibiotics: 100 U/mL penicillin and 100 µg/mL streptomycin, at 37 °C in a humidified atmosphere with 5% CO_2_. The cell culture was supplemented with the fresh growth medium two or three times per week. The cell count and viable cell number were assessed using a standard trypan blue exclusion method with a hemocytometer. To obtain a cell suspension for the cytotoxicity testing or to maintain the cell culture, sub-confluent cultures (70–80%) were treated with 0.25% trypsin and seeded at a density of 0.5–3 × 10^4^ cells/cm^2^.

The extract (see Supplementary Method online) and direct tests were performed to evaluate the biocompatibility of the biomaterials. L929 fibroblasts were seeded at a density of 1 × 10^4^/cm^2^ and allowed to grow for 24 h. Then the medium was replaced with the fresh growth medium supplemented with autoclaved biomaterial at a final concentration from 0 to 10%. The cells were further cultured for 24 or 72 h. To prepare the films for in vitro cell culture studies, the biomaterial solution was pipetted onto 96- or 24-well plates wells and left to dry under sterile conditions. After 72 h, the films were washed with sterile 0.1 M NaOH for 10 min (to remove residual acetic acid), followed by five washes with sterile distilled water.

The films prepared on 96-well plates were submerged with the growth medium and incubated for 3 h at 37 °C in a humidified atmosphere with 5% CO_2_. Further, L929 fibroblasts were seeded on the films at a density of 1 × 10^4^/cm^2^ and allowed to grow for 24 and 72 h. The effect of the biomaterials on the L929 cell viability was assessed using the MTT assay, which is based on the reduction of 3-(4,5-dimethylthiazol-2-yl)-2,5-diphenyltetrazolium bromide (MTT) by mitochondrial dehydrogenases of viable cells. At the end of the experiment (24 or 72 h treatment), the medium was removed, and 100 µL of 0.5 mg/mL MTT (Sigma-Aldrich) prepared in the fresh growth medium was added to each well. After 3 h of incubation at 37 °C in a humidified atmosphere with 5% CO_2_, the solution was removed, 100 μL of 2-propanol with 0.04 N HCl was added to each well to dissolve the produced purple formazan crystals, and the plates were shaken for 15 min at 100 rpm. Then, the absorbance was measured at a wavelength of 570 nm with background subtraction at 690 nm using a microplate reader (Multiskan Spectrum; Thermo Scientific, Waltham, MA, USA). The number of viable cells was calculated relative to the control cells growing in the growth medium without the biomaterial (direct test) or its extract (extract test). The experiments were performed at least in triplicate.

#### Statistical analysis

The data are expressed as mean ± standard error of the mean. The results were obtained from at least three replicates. One-way analysis of variance (ANOVA) followed by a Dunnett or Tukey post hoc test for multiple comparisons was used to determine the statistical significance. Grouped comparisons were performed using two-way ANOVA followed by Tukey’s multiple comparisons test. Statistical analysis and data visualization were performed with GraphPad Prism 9.2 software (GraphPad Software, San Diego, CA, USA). A *p*-value of less than 0.05 was considered statistically significant.

## Results and discussion

### Characterization of the obtained films

CBD oil comes in different concentrations and can be applied directly to the skin. Thanks to the main natural active ingredient in the oil (cannabidiol), its anti-inflammatory and antioxidant properties are increased tenfold; therefore, the skin regenerates more quickly^36^. Besides cannabidiol, the oil also contains a wide range of other beneficial organic compounds such as phenols, terpenes, flavonoids, and an ideal combination of omega-3, omega-6, and omega-9 fatty acids, which occur naturally. Combining all these compounds in the oil with a natural, non-toxic, and biodegradable polysaccharide might positively affect wound healing, hence the choice of designing and obtaining the novel chitosan-based biomaterials containing cannabis oil (CBD-CS, 20% concentration) with high cannabidiol content. Three sample films were prepared by setting the mass concentration of CBD: 1CBD-CS, 5CBD-CS, and 10CBD-CS. The thickness of which films was 0.0499 mm, 0.0521 mm, and 0.0517 mm, respectively. The diameter of all the obtained films was 55 mm.

**Figure 1:**
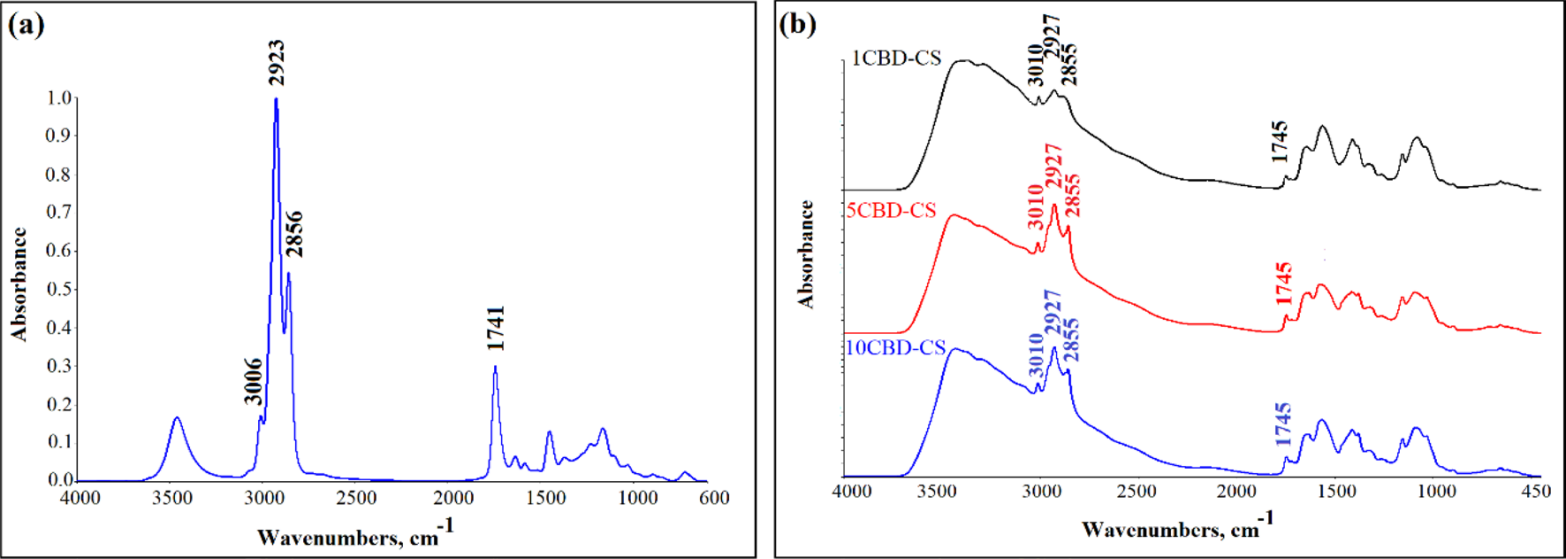
FTIR spectrum of the (**a**) cannabis oil and (**b**) CBD-CS films; cannabidiol-chitosan-based films (CBD-CS), where 1, 5, 10CBD-CS corresponds to the weight ratio of 1, 5 or 10% of cannabidiol oil (CBD) to chitosan (CS).

The chemical structure of the pure chitosan, CBD oil, and all obtained films was tested by ATR-FTIR spectroscopy (Fig. 1). According to the literature^46^, the spectrum of pure chitosan (see Supplementary Fig. S2 online) shows absorption bands attributed to OH stretching (2900–3500 cm^−1^), CH stretching (2860–3100 cm^−1^), NH bending (1560 cm^−1^), OH and CH deformation vibrations (1408, 1385 cm^−1^), and C–O–C stretching vibrations (1000–1260 cm^−1^). For CBD oil, the band at 3458 cm^−1^ corresponds to the stretching vibration of the OH groups of cannabinoids. The band at 3006 cm^−1^ was assigned to the CH stretching of benzene rings. Two bands at 2923 cm^−1^ and 2856 cm^−1^ are the stretching vibrations of the CH_3_ and CH_2_ groups of cannabinoids and fatty acids hydrocarbons. The 1741, 1634, 1582, and 1449 cm^−1^ bands were assigned to the benzene skeleton vibrations. The band at 1368 cm^−1^ is the bending vibration of CH_3_, and the 1224 cm^−1^ band was assigned to CO stretching vibration^47^. After CBD was embedded in chitosan films, the characteristic bands of cannabis oil at 3010, 2927, 2855, and 1745 cm^−1^ in all samples were observed. The spectrum of the obtained films also shows bands characteristic of chitosan, such as a broad, intense band at 3426 cm^−1^ or a band at 1562 cm^−1^ associated with its amino groups. Some bands are shifted due to the intermolecular interaction between CBD and chitosan. The IR spectra indicated that CBD oil was successfully incorporated into the chitosan films.

**Figure 2:**
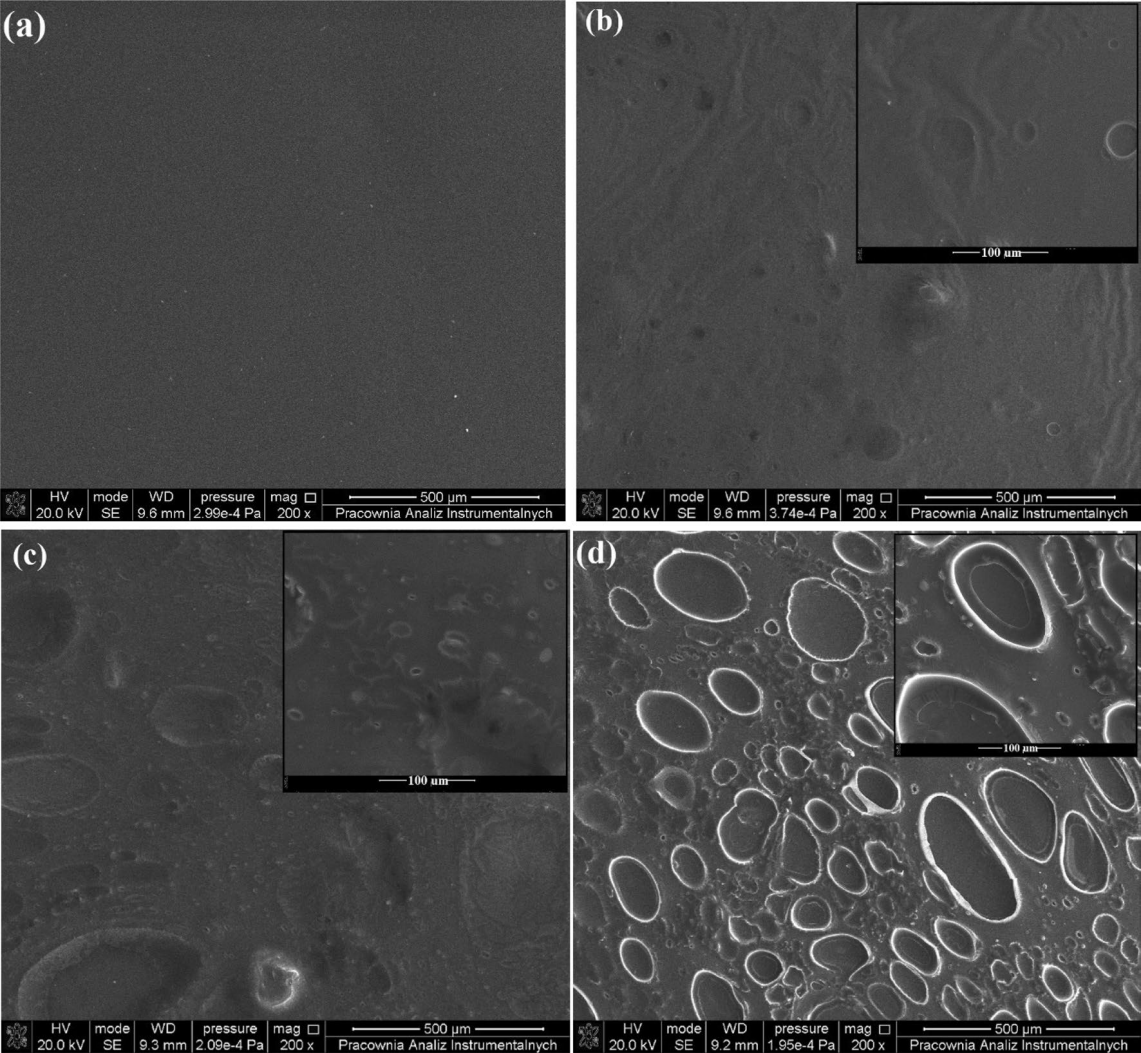
SEM images of (**a**) chitosan, (**b**) 1CBD-CS, (**c**) 5CBD-CS, and (**d**) 10CBD-CS films; cannabidiol-chitosan-based films (CBD-CS), where 1, 5, 10CBD-CS corresponds to the weight ratio of 1, 5 or 10% of cannabidiol oil (CBD) to chitosan (CS).

The surface morphology of CBD-CS films was studied using Scanning Electron Microscopy^48^. Figure 2a illustrates the SEM image of pure chitosan film, which has a smooth surface and does not form any clusters or cracks. The presence of CBD oil in the obtained CBD-CS films impacts the material morphology, as presented in Fig. 2b–d. The oil appears as scattered spherical shapes embedded in the chitosan layer. The increasing CBD content in the samples showed a gradual increase in the size of these shapes. Similar results were reported by da Silva Fereira et al.^49^ with chitosan film containing andiroba oil, demonstrating ellipsoidal-shaped structures correlated with forming two phases in the matrix (lipid and polymer).

**Figure 3:**
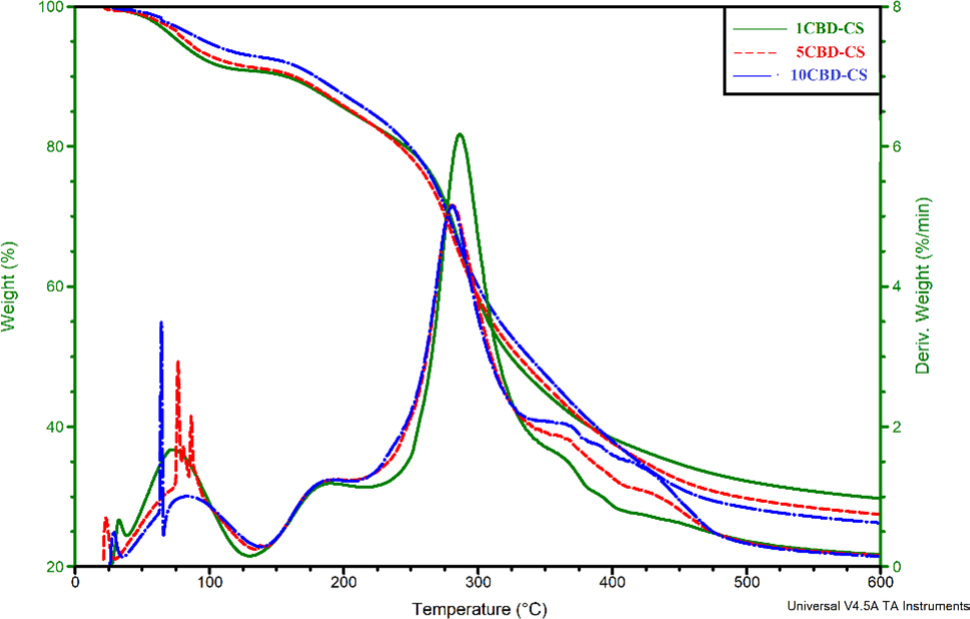
Thermal analysis of the CBD-CS films; cannabidiol-chitosan-based films (CBD-CS), where 1, 5, 10CBD-CS corresponds to the weight ratio of 1, 5 or 10% of cannabidiol oil (CBD) to chitosan (CS).

The thermal stability of the samples was studied using thermogravimetric analysis (TGA). The observation of different stages of degradation was performed using differential thermogravimetric analysis (DTG). The analysis was performed at room temperature to 600 °C in a nitrogen atmosphere. The results obtained for pure chitosan film and all cannabis oil-containing chitosan-based films are presented in Fig. 3 and listed in Table S1 (see Supplementary Table S1 online). As presented in Fig. 3, the TG curve shows three stages of the thermal decomposition of chitosan. The first occurs at 20–120 °C, corresponding to about 10% mass loss, and is related to the release of water adsorbed by the polymer, whereas the maximum loss rate occurs at 69 °C. The second and third stages, associated with the decomposition of chitosan, start at about 128 °C and 222 °C, respectively, with a mass loss of 7% and 55% being associated with breaking polysaccharide chains (including dehydration, deamination, deacetylation, breakage of glycosidic bonds, and opening of the pyranose ring) and elimination of small-molecule degradation products. All chitosan-based films incorporated with cannabis oil, similarly to chitosan film, underwent three degradation stages. After CBD was embedded into chitosan films, the thermal stability of all CBD-CS films remained high but slightly decreased compared to the pure chitosan film. It is probably because of the lower decomposition temperature of the pure CBD. It was observed that the lower thermal stability was associated with the increased concentration of oil in the polymer films. However, all the obtained materials show good stability at higher temperatures, beneficial for biomedical applications.

The mechanical properties of the new biomaterials have an impact on their potential application. Wound dressing materials should express appropriate mechanical properties to avoid possible injuries to the wound. As shown in Table 1, the mechanical properties of 1CBD-CS, 5CBD-CS, and 10CBD-CS were significantly enhanced compared with pure chitosan. Furthermore, Young's modulus and elongation at the break of the CBD-CS samples were significantly improved with increasing CBD oil content. In the case of 10CBD-CS, this modulus is 350% greater than pure chitosan. A similar phenomenon was reported in the lemongrass oil encapsulated in starch–alginate film, where the percent elongation at break increased as the concentration of lemongrass oil increased^50^. In another study incorporating the *Acalypha indica* extract onto the polymeric blend also increased the tensile strength property^51^. The best tensile strength was obtained for the 5CBD-CS sample at 3.61% elongation. The slight decrease in tensile strength of the 10CBD-CS sample may be related to the higher oil content that, as shown in Fig. 2, results in the loosening of the film structure. Despite this, all the designed films revealed good mechanical properties, which allows their potential use as wound dressings.

**Table 1:** Mechanical properties of the obtained materials.

Sample	Mechanical properties		
	Tensile strength (MPa)	Elongation (%)	Young’s modulus (MPa)
CS	4.23 ± 0.12	3.78 ± 0.06	80.74 ± 0.27
1CBD-CS	5.67 ± 0.06^a^	2.82 ± 0.05^a^	185.47 ± 0.31^a^
5CBD-CS	12.35 ± 0.03^a,b^	3.61 ± 0.02^b^	299.41 ± 0.81^a,b^
10CBD-CS	9.68 ± 0.05^a,b,c^	3.49 ± 0.09^a,b^	367.30 ± 0.52^a,b,c^

CBD-CS—cannabidiol-chitosan-based films, where 1, 5, 10CBD-CS corresponds to the weight ratio of 1, 5 or 10% of cannabidiol oil (CBD) to chitosan (CS).

^a,b,c^Indicate *p* < 0.05 when compared to the corresponding CS, 1CBD-CS and 5CBD-CS, respectively.

**Figure 4:**
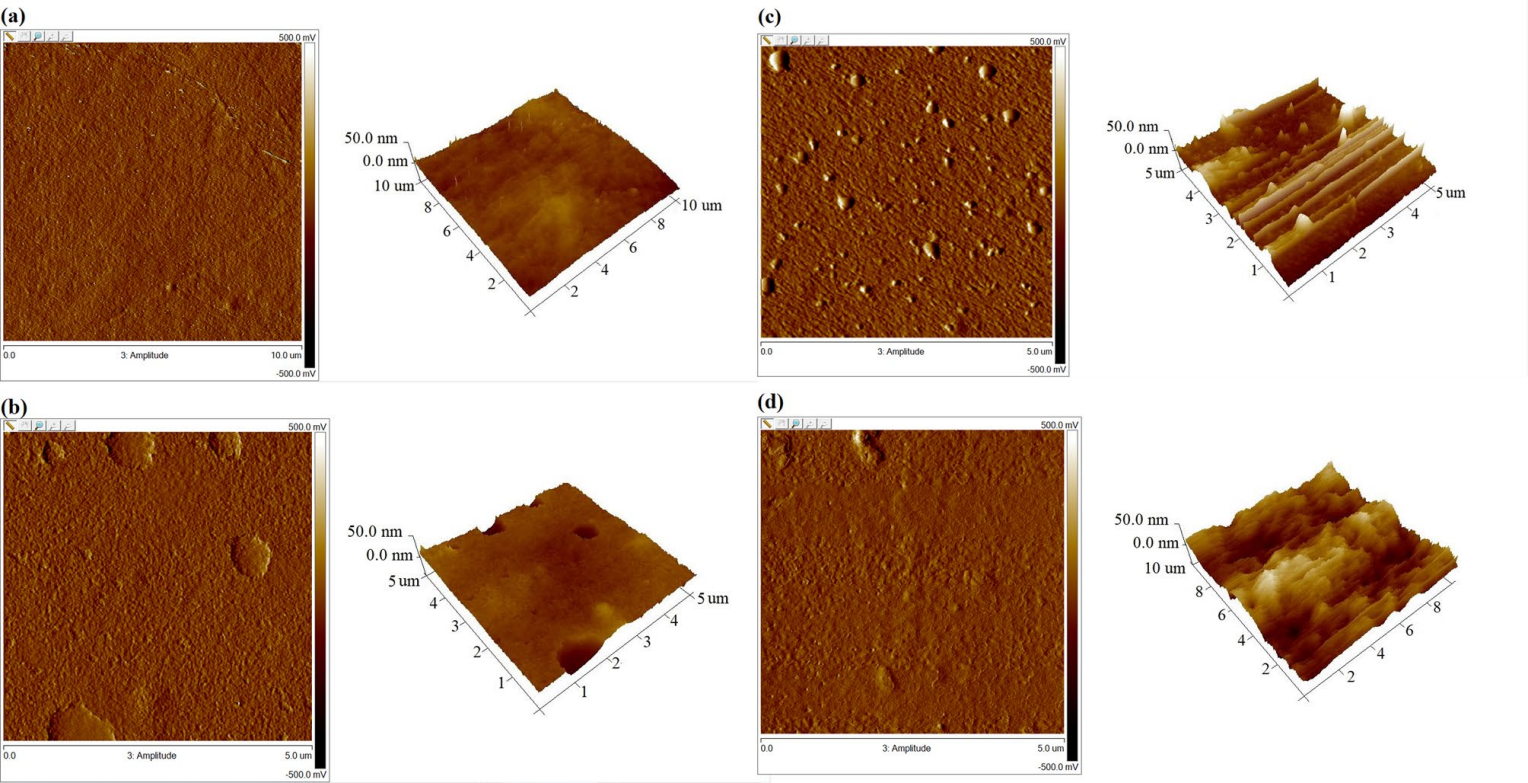
AFM images of (**a**) chitosan, (**b**) 1CBD-CS, (**c**) 5CBD-CS, and (**d**) 10CBD-CS films). CBD-CS—cannabidiol-chitosan-based films, where 1, 5, 10CBD-CS corresponds to the weight ratio of 1, 5 or 10% of cannabidiol oil (CBD) to chitosan (CS).

Topographic atomic force microscopy (AFM) is used to evaluate the morphology of samples and surfaces as well as to obtain topographic images and the roughness of scanned compounds. The AFM images of the obtained materials are shown in Fig. 4, and their roughness parameters are summarized in Table 2. The pure chitosan film shows a relatively smooth surface morphology with a maximum roughness (R_max_) of 45.8 nm. Along with incorporating of cannabis oil, the surface of the resulting biomaterials becomes rougher. The sample with the highest oil concentration (10CBD-CS) reveals the roughest surface, with a maximum roughness of 441 nm. The rough topography of all films may be advantageous for cell adhesion and crucial for dermal and epidermal tissue regeneration^52^. The authors' previous studies^53–57^ revealed that the rough surfaces of the biomaterials are suitable for accelerating cell adhesion, proliferation, and skin regeneration. The rough surfaces display a high surface area, improving cell attachment, which is beneficial for their antimicrobial wound dressings and as tissue regeneration scaffolds to induce the adhesion of dermis fibroblasts. Additionally, the rough structure might enhance the adsorption of biological fluids, including erythrocytes, that might improve the hemostatic ability of potential wound dressing^58^. However, the literature shows that the response of cells to roughness varies depending on the cell type^59–61^. Zhong et al.^62^ obtained electrospun collagen nanofibrous scaffolds, showing reduced adhesion of rabbit conjunctival fibroblasts to smoother surfaces. The authors attributed this phenomenon to reduced surface interaction when the cells first come into contact with the surface. Therefore, a rougher biomaterial surface could be beneficial for cell attachment, as it would offer more surface area for initial cell attachment. Nevertheless, the surface roughness values obtained for the CBD-CS samples are suitable for cell attachment and growth in tissue engineering applications, consistent with the literature data^63–65^.

**Table 2:** Surface characterization of the obtained materials.

	SAMPLE			
	CS	1CBD-CS	5CBD-CS	10CBD-CS
**Roughness parameters [nm]**				
R_q_	4.15 ± 0.01	4.98 ± 0.01^a^	9.73 ± 0.01^a,b^	63.4 ± 0.20^a,b,c^
R_a_	3.25 ± 0.01	3.73 ± 0.01	6.73 ± 0.01^a,b^	46.8 ± 0.30^a,b,c^
R_max_	45.8 ± 0.12	46.30 ± 0.06	135 ± 0.31^a,b^	441 ± 0.56^a,b,c^
**Average contact angle [θ, °]**				
Water	93.6 ± 0.05	78.3 ± 0.05^a^	79.5 ± 0.07^a,b^	77.1 ± 0.05^a,b,c^
Diiodomethane	55.7 ± 0.04	20.5 ± 0.07^a^	13.6 ± 0.03^a,b^	22.7 ± 0.03^a,b,c^
**Surface free energy [mJ/m**^**2**^**]**				
γ_s_	30.57 ± 0.29	47.03 ± 0.14^a^	48.63 ± 0.32^a^	46.51 ± 0.21^a,c^
γ_s_^d^	28.70 ± 0.23	43.27 ± 0.05^a^	45.64 ± 0.24^a,b^	42.12 ± 0.51^a,c^
γ_s_^p^	1.87 ± 0.03	3.76 ± 0.03^a^	2.98 ± 0.15^a,b^	4.39 ± 0.03^a,b,c^

CBD-CS—cannabidiol-chitosan-based films, where 1, 5, 10CBD-CS corresponds to the weight ratio of 1, 5 or 10% of cannabidiol oil (CBD) to chitosan (CS).

^a,b,c^Indicate p < 0.05 when compared to the corresponding CS, 1CBD-CS and 5CBD-CS, respectively.

The contact angle measurement is essential in evaluating the hydrophilicity/hydrophobicity of the sample. It provides information on the structure of the surface and its wettability. The obtained contact angle results with water and diiodomethane measurements are shown in Table 2, and the contact angle with glycerin results are given in Supplementary Table S2 online. As can be seen, the contact angle for pure chitosan with water was 93.6° ± 0.05, indicating its hydrophobic nature. On the other hand, the contact angles of chitosan-based films containing the cannabis oil were significantly less than those of pure chitosan, which indicates a more hydrophilic nature of the obtained biomaterials (see Supplementary Figure S3 online). Sedlarikova et al.^66^ also found that adding of *Andiroba* oil to chitosan confers more excellent hydrophilicity to the material. The sample with the highest CBD oil content exhibited the most wettable surface, which is beneficial because the hydrophilic nature of the materials is strongly favored in wound dressing design. A hydrophilic dressing maintains a moist environment, high exudates or blood adsorption, and high water vapor permeability at wound sites^67^. Moreover, higher wettability films could enhance the release of natural substances, as demonstrated by the HPLC analysis (see Supplementary Figure S6 online).

**Figure 5:**
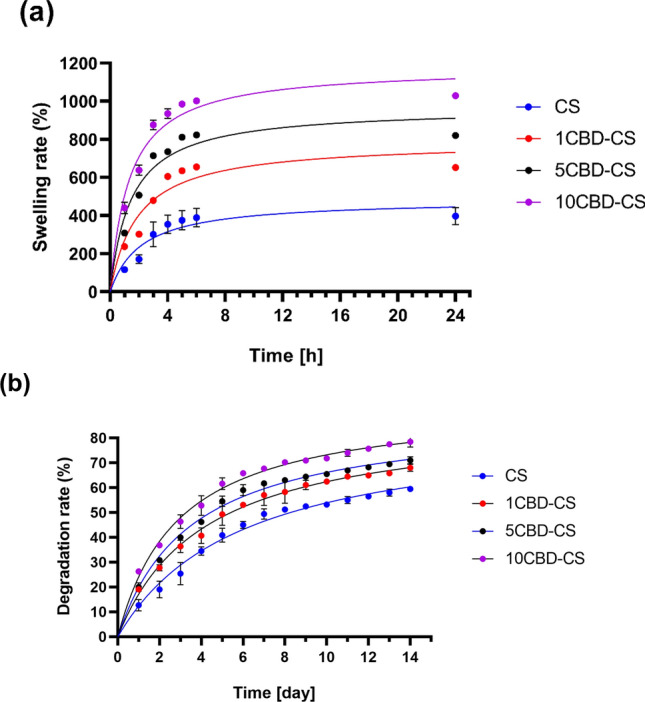
(**a**) Swelling and (**b**) degradation studies of the obtained films, where 1, 5, 10CBD-CS corresponds to the weight ratio of 1, 5 or 10% of cannabidiol oil (CBD) to chitosan (CS).

Evaluation of the swelling and degradation ratios is essential in wound dressing design. A high swelling rate can reduce the risk of skin damage around the wound and scar formation^68^. The swelling behavior of the chitosan-based films enriched with cannabis oil was tested by immersing them in PBS solution for up to 24 h. After 1, 2, 3, 4, 5, 6, and 24 h, the samples were removed from the solution and weighed. The obtained results are shown in Fig. 5a. As can be seen, all the obtained films exhibit good swelling ability. Compared with pure CS film, adding oil significantly increased the liquid absorption capacity of the films. Moreover, as the cannabis oil concentration increased, the swelling of the samples increased. Such a phenomenon has also been reported elsewhere^69–71^. After 24 h, the swelling ratios of 1CBD-CS, 5CBD-CS and 10CBD-CS were 662.9%, 836.6% and 1029.4%, respectively. In the 5–24 h, the swelling ratios increased slightly, suggesting that prolonged immersion does not affect the swelling of the obtained biomaterials. Moreover, the swelling ability is related to hydrophilicity, confirmed by the wetting angle values of the obtained films. The 10CBD-CS film has the highest swelling index, exhibiting the most hydrophilic character among all obtained biomaterials. All chitosan-based films containing cannabis oil have good swelling behavior, indicating that they could absorb wound exudate and accelerate wound healing. Moreover, the high amount and fast swelling rate suggest that the obtained dressings could rapidly release active substances (in this case, cannabidiol) to the wound^72^.

Degradation analysis is another crucial factor in wound dressing design. All cannabis oil-chitosan-based films were placed in PBS solution, and the degradation was monitored for 14 days (Fig. 5b). Both pure chitosan and all the obtained biomaterials were found to decompose, and the degradation rate of the samples increased gradually with time. It can be seen that the films containing cannabis oil exhibited a higher degradation rate as compared to pure chitosan. As the concentration of oil in the films increased, the degradation of the samples increased, which may be due to the presence of partially biodegradable cannabidiol but also due to the more hydrophilic properties and more accessible access of water to the film components^73^.

### Water vapor transmission rate (WVTR)

Another important factor in wound dressing design is the optimal water vapor transmission rate (WVTR). For healthy skin, this parameter is about 0.85 mg/cm^2^/h^74^. Lower vapor transmission rates of dressing materials compared to healthy skin values increase the risk of wound infection. It is assumed that an ideal wound dressing should demonstrate the WVTR range between 8.33 and 10.42 mg/cm^2^/h to allow wound healing^67^. The determined WVTR values for the studied films are not within the desired range (Table 3). However, the chitosan film with the highest cannabis oil content, has a WVTR value relatively close to the desired range, and all samples have higher WVTR values than those expressed by healthy skin. Therefore, the obtained films reveal the potential to be used as a base material in dressing.

**Table 3:** The properties determined for the obtained films.

Sample	Release of CBD (%)	DPPH scavenging (%)	Oxygen permeability (mg/L)	Water vapor transmission rate (g/m^2^/h)
CS	–	9.5 ± 0.64	8.71 ± 0.01	3.21 ± 0.02
1CBD-CS	8	42.5 ± 0.25^a^	8.91 ± 0.003^a^	4.16 ± 0.02^a^
5CBD-CS	19	64.8 ± 0.36^a,b^	9.12 ± 0.01^a,b^	5.90 ± 0.01^a,b^
10CBD-CS	7	72.7 ± 0.05^a,b,c^	9.22 ± 0.003^a,b,c^	7.37 ± 0.01^a,b,c^

CBD-CS—cannabidiol-chitosan-based films, where 1, 5, 10CBD-CS corresponds to the weight ratio of 1, 5 or 10% of cannabidiol oil (CBD) to chitosan (CS).

^a,b,c^Indicate *p* < 0.05 when compared to the corresponding CS, 1CBD-CS and 5CBD-CS, respectively.

In general, the incorporation of plant extracts into polysaccharide films causes an increase in water vapor permeability through the films, as confirmed by other researchers^75–77^. It is likely related to the interaction of the oil components with the polymer chain, causing an increase in interfacial interaction between the matrix and the extract. This phenomenon discourages interaction between polymer chains and water molecules; consequently, the WVTR parameter was increased.

### Oxygen permeability

Adequate oxygen supply to the wound is essential for optimal healing and reducing the risk of infection. Therefore, it is essential to study their oxygen permeability when designing new dressing materials. The results from the analysis of oxygen permeability across chitosan-based films incorporated with cannabis oil are shown in Table 3. The amount of dissolved oxygen in the negative control and positive control water was 7.50 and 11.84 mg/L, respectively, whereas in the samples collected from bottles covered with pure chitosan, 8.71 mg/mL. The films with cannabis oil had higher dissolved oxygen values than pure chitosan. The oxygen permeability rose with the oil content increase and revealed the highest oxygen value of 9.22 mg/mL for the 10CBD-CS film. Similarly, Khaliq et al.^69^ studied oxygen penetration using the Winkler method. They reported that the oxygen amount dissolved in water was 7.2–11.6 mg/mL for the chitosan-based membranes enriched with κ-carrageenan. The values obtained for the films in our study (about 8.71–9.22 mg/mL) can be considered sufficient for a wound dressing material. Moreover, these results indicate that all the obtained biomaterials allow good oxygen permeation, essential in biomedical applications to promote cell regeneration and accelerate wound healing.

### Antioxidant effects

A good dressing material should have antioxidant properties that promote wound healing by scavenging overproduced free radicals^78^. The DPPH assay is the standard method for detecting materials' free radical scavenging activity. According to the obtained results (Table 3), the pure chitosan film showed a poor scavenging ability of only 9.5%. Incorporating cannabis oil into the chitosan matrix significantly improved the scavenging activity. As the oil concentration increased, the degree of DPPH free radical scavenging by the obtained biomaterials enhanced to 42.5%, 64.8%, and 72.7% for 1CBD-CS, 5CBD-CS, and 10CBD-CS films, respectively. Similar results were also reported by Bölgena et al.^79^ who introduced *Hypericum perforatum* (HP) oil to chitosan cryogel and determined the antioxidant activity of the samples using a DPPH assay. The authors found that the degree of DPPH free radical scavenging increased with the increasing HP oil content in chitosan cryogel. Abbas et al.^80^ reported that the enhanced antioxidant activity of chitosan-polyvinyl alcohol membranes was accompanied by the increasing concentration of the *Calotropis procera* extract. These outcomes also showed good similarity to our findings.

The results show that CBD oil exhibits strong antioxidant properties. The oil's main component, cannabidiol, is its antioxidant potential^81^. Hence, the obtained CS-CBD films could be regarded as potential agents for wound dressing.

### Release of CBD from the obtained materials

In the case of drug-containing dressings, the release rate of active substances needs to be adjusted to provide effective treatment. Therefore, the release of the active ingredient from the obtained chitosan films was performed to evaluate its further suitability for wound healing. The cannabidiol release from the materials as a function of time is presented in Supplementary Figure S4 and Table S4 online, and the percentage of total CBD release is shown in Table 3. In addition, the HPLC chromatograms are given in Supplementary Figure S6 online. The cannabidiol release was monitored at the indicated time points over an 85 h period. Cannabidiol was released with the highest rate from the 5CBD-CS sample; approximately 13% of CBD was released after only 40 min. These results suggest that this sample could be potentially used as a drug carrier in rapid drug release applications. From other materials, the gradual release of CBD was noted. It is also worth noting that after the initial rapid release phase, the release of cannabidiol from the chitosan matrix occurs in a delayed manner, which is a significant observation when designing controlled drug delivery systems. Khan et al.^82^ also initially observed immediate release of the natural active ingredient from poly(vinyl)alcohol/chitosan films, followed by a sustained release. This quick release of the natural active ingredient from the film may be due to the high drug concentration. After some time, the release of the drug becomes slower due to the lower concentration of the drug in the film and higher concentration in the medium^83^. However, we found no relationship between the amount of released active ingredient and its concentration. The highest amount of cannabidiol (19%) was released in the 5CBD-CS sample, which most likely means that CBD did not mix evenly with chitosan; hence it might be evidence of the oil presence near the film surface. In the other samples, the cannabis oil quickly penetrated the cross-linked structure of the chitosan, causing the hydroxyl and amino groups to face towards the surface. The polar component result supports this assumption, 2.98 ± 0.15 mJ/m^2^ for 5CBD-CS, effectively displaying the most hydrophilic behavior compared with the other samples. In addition, the SEM images show that the oil is present on the sample surface and is not incorporated into the chitosan network structure, as observed in the images of the other samples. The results indicate that all the materials incorporated with natural active substances can enhance wound healing compared to dressings without this drug.

### Protein adsorption

**Figure 6:**
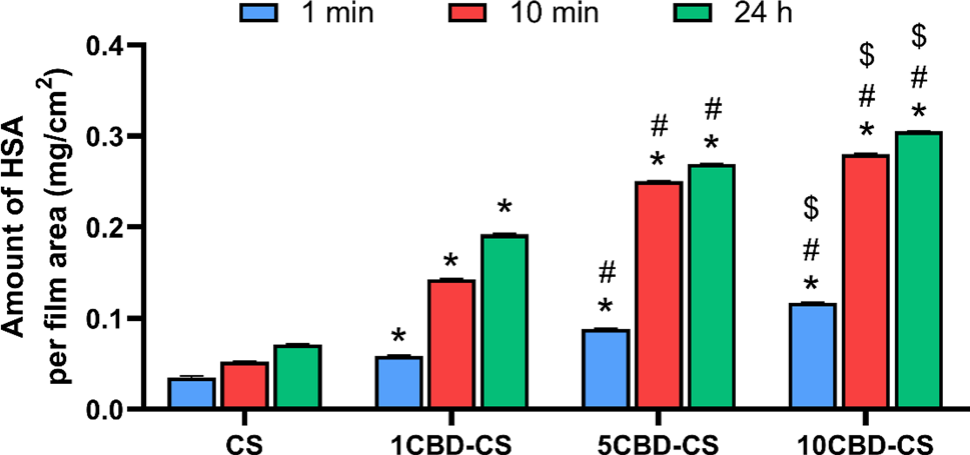
Amount of bounded human serum albumin at the surface of the obtained films, where 1, 5, 10CBD-CS corresponds to the weight ratio of 1, 5 or 10% of cannabidiol oil (CBD) to chitosan (CS). ^*, #, $^ indicate *p* < 0.05 when compared to the corresponding CS, 1CBD-CS and 5CBD-CS, respectively.

When designing wound dressings, it is essential to evaluate the adsorption capacity of proteins, especially plasma proteins, at the solid–liquid interface of the biological fluid and the wound dressing material. When blood is in contact with external materials, plasma proteins first adsorb over their surface, further directing the adhesion of morphotic blood elements^84^. Albumin is the most abundant plasma protein found in the blood. Therefore, human serum albumin was used to assess the interaction with the obtained cannabis oil-containing chitosan-based films. The amount of bound proteins with the obtained materials was measured spectrofluorimetrically after different incubation times (1 min-24 h). The results are shown in Fig. 6. A table and figure containing the whole time range are given in Supplementary Table S3 and Figure S5 online, respectively. All the biomaterials show the capability to interact with HSA. It has been noted that the human serum albumin adsorption capacities of 1CBD-CS, 5CBD-CS, and 10CBD-CS increased with increasing cannabis oil concentration. The protein adsorption amounts had a sudden increase in the first stage, and after only 10 min of incubation, all CBD-containing materials bound about two times more protein. After about 15 min, the adsorption slowed down, and a minor increase in the amount of HSA bound to the films was observed. It is worth noting that protein adsorption on the 10CBD-CS sample after 24 h is about three times higher than on pure chitosan. In comparison, Awadhiya et al.^72^ after 20 min of incubation, rendered only 180 ng/cm^2^ of bovine serum albumin-bound on agarose bioplastic. Moreover, those authors have argued that “surfaces that strongly adsorb proteins will bind cells”^72^. Furthermore, the protein adsorption property of a wound dressing is closely related to its cell adhesion behavior. Atomic force microscopy studies confirmed that higher sample roughness promotes cell adhesion. The most protein bound to the surface revealed the 10CBD-CS sample, which also presents the roughest texture. Furthermore, the polar component of this material has the highest value; thus, this sample interacts very well with the protein. The oil containing chitosan-based films might be potentially applied in wound dressing.

### Microtox test

The prepared films were subjected to an acute toxicity test using Microtox. The test is based on the decrease of the bioluminescence of the Gram-negative *Aliivibrio fischeri* bacteria at 490 nm^85^ after exposure to a tested sample, which is correlated with the metabolism and cell viability^86^. As the *A. fischeri* species are Gram-negative bacteria, the antibacterial activity exerted by the sample was found to decrease the cell viability of the bacteria^87–89^, which led to the possibility of the initial assessment of the antibacterial activity of the tested samples^90^. The results may sometimes be exaggerated due to the test's specificity, wherein the bacteria used are susceptible to enable rapid measurements^91^.

**Figure 7:**
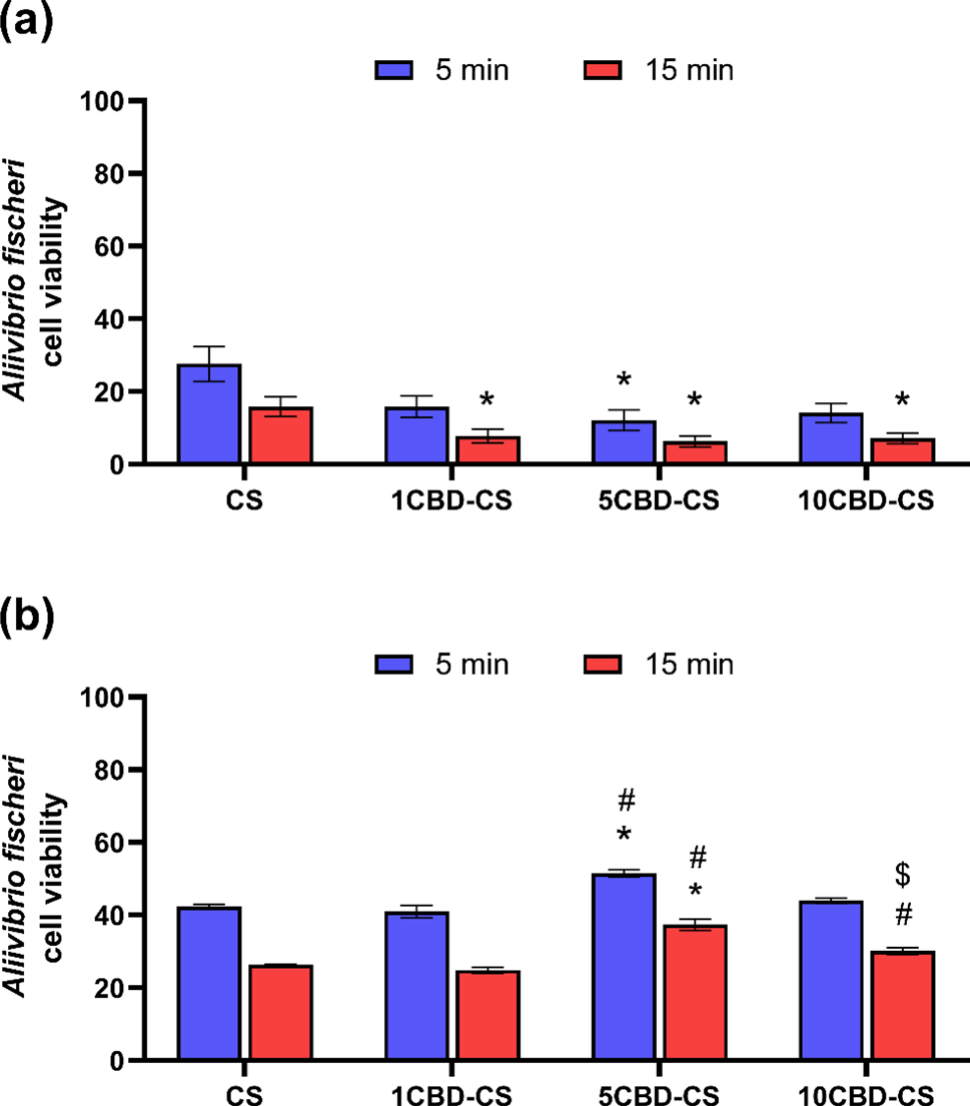
Changes in the *A*. *fischeri* viability after 5 and 15 min upon exposure to (**a**) the intact fragments of the prepared films and (**b**) the film's suspension in water. 1, 5, 10CBD-CS corresponds to the weight ratio of 1, 5 or 10% of cannabidiol oil (CBD) to chitosan (CS). ^*, #, $^ indicate *p* < 0.05 when compared to the corresponding CS, 1CBD-CS and 5CBD-CS, respectively.

Two experiments were performed to mimic the potential use of the prepared films in wound dressing materials. The films were placed in direct contact with the bacteria, which allowed to simulate the application of the film to the infected tissue (Fig. 7a). In a separate experiment, the films were first dispersed in water, and the resulting suspensions were applied to the test, thus imitating the infection combined with wound exudate (Fig. 7b). In both cases, the cell viability was determined by comparison with the bacteria treated with medium (2% saline) alone.

As can be seen, by the drop in *A. fischeri* cell viability, both experiments suggest the prepared films' acute toxicity. On the one hand, this is probably the result of the presence of chitosan, which presents strong antimicrobial properties^92^. On the other hand, the antimicrobial activity of cannabinoids is also known^93^. Both of these materials are known for their high biocompatibility^94,95^, suggesting that the observed toxic effect might be due to the antimicrobial activity of the films exerted on the *A.* *fischeri* bacteria. Overall, the effect exerted by the intact films was greater than that of the suspensions. The addition of cannabis oil into the CS film potentiates its activity. Notably, the increase in the cannabis oil content in the film significantly decreases the viability of *A. fischeri* if the films are in direct contact with the bacteria. Quite the opposite can be observed in the case of dispersed film suspensions. The cell viability increases along with the extract concentration in the films, suggesting the protective effect of the cannabinoid extract on bacteria. What should be noted is that although the concentration of film components in the simulated exudate was 100 times lower, the exerted toxic effect was also lower but not linear to the dilution and still significant (cell viability below 30% of that of the negative control). Such a phenomenon could probably be due to the formation of smaller particles that have easier access to the bacteria or an instant effect of the antimicrobial compounds, which did not need to be released from the film. In the case of the simulated exudate experiment, the observed effect diminishes with the concentration of the cannabinoids up to a certain point (10% w/v), after which the effect starts to rise again. In terms of the potential use of the prepared films as the dressing materials, such results would suggest that the prepared films could efficiently prevent any bacterial infection of the wound.

### Biocompatibility evaluation using L929 mouse fibroblasts

**Figure 8:**
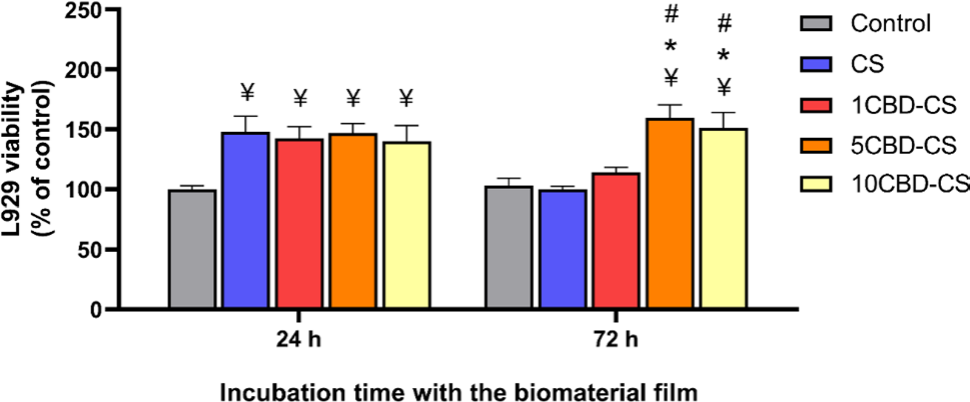
Cytotoxicity evaluation in L929 fibroblast cell culture. The viability of L929 cultured with the prepared films for 24 and 72 h (direct test). The results are presented as the percentage of the cell viability compared to the control cells cultured in the growth medium only for the indicated time point. ¥, *, # indicate *p* < 0.05 when compared to the corresponding Control, CS and 1CBD-CS, respectively.

In vitro cytotoxicity testing is an indispensable part of developing biomaterials. To evaluate the potential of the designed CBD-enriched chitosan membranes for wound dressing application, we studied their indirect and direct effects on the viability of L929 mouse fibroblasts (Fig. 8). Following ISO standard 10993-5, two different culturing methods were implemented to evaluate if there is a cytotoxic response to the tested materials: direct contact and extract tests. The results of the extract test where L929 cells were grown in the control growth medium or the extract of the tested biomaterials are presented in Supplementary Figure S6 online. As reported by^96–98^, the chitosan (CS) extract increased cell viability after 24 and 72 h exposure time. For 24 h treatment, chitosan materials enriched with CBD exerted similar beneficial effects as the pure chitosan (increased viability by approx. 50% as compared to the control cells grown in the growth medium only). Interestingly, the viability of L929 fibroblasts cultured in the CBD-CS extracts for 72 h was comparable to the control cells. To further evaluate the biocompatibility of the CBD-CS materials, L929 fibroblasts were cultured with the prepared films to determine their direct effect on cell viability (Fig. 8). For 24 h treatment, similar to the extract test results, CS and CBD-CS films increased the cell viability by approx. 50% as compared to the control cells. We found no substantial differences between the CS and the CBD-enriched chitosan. However, for the more prolonged time exposure to the films (72 h), a significant increase was observed in the cell proliferation in the presence of 5CBD-CS and 10-CBD-CS, as compared to the pure chitosan. Notably, the cells cultured with the CS films for 72 h exhibited comparable viability as the control cells. This suggests that CBD, particularly at concentrations of 5 and 10%, exerts beneficial long-term effects. However, the highest increase in cell proliferation was observed for the 5CBD-CS film, which may be related to the surface free energy results. The 5CBD-CS film exhibited the highest surface free energy (48.63 ± 0.32 mJ/m^2^) compared with the other samples. As reported in other works^99,100^, surfaces with higher surface free energy promote cell adhesion and proliferation, while substrates with low surface energy tend to inhibit cell adhesion.

The results indicate that the CBD-enriched chitosan materials are non-toxic to the mouse fibroblasts and may constitute promising biocompatible support for further wound dressing design.

## Conclusions

Developing novel biomaterials for wound dressing applications using natural polymers and active substances with antimicrobial properties is beneficial for wound treatment. Cannabis oil is rich in many active compounds, and its main component—cannabidiol, provides antimicrobial, antioxidant, regenerative, and antithrombotic properties of beneficial properties for wound treatment. The current study presents the preparation and characterization of novel natural chitosan-based films incorporating cannabis oil for wound dressing. All chitosan-based film samples with cannabis oil exhibited good mechanical and hydrophilic properties and a high swelling ratio. Degradability tests showed that the films lost their maximum weight within eight days. Moreover, increased concentration of cannabis oil in the samples promoted adsorption of human albumin. The obtained biomaterials showed good antimicrobial activity against *A. fischeri* and a good drug release profile. In addition, cell culture studies demonstrated that designed CBD-CS biomaterials are biocompatible. Therefore, chitosan-based films incorporating cannabis oil might be an excellent candidate for wound treatment and dressing.

## Supplementary Information

Supplementary Information.

## Data Availability

The datasets generated and analyzed during the current study are available from the corresponding author on reasonable request.
