# Supplementary material for: Chitosan-based films with cannabis oil as a base material for wound dressing application
Source: Sci Rep. 2022 Nov 4;12:18658. doi: 10.1038/s41598-022-23506-0 (PMC9636169; doi:10.1038/s41598-022-23506-0)
Supplement: Supplementary file 1 — Supplementary Information. [file 41598_2022_23506_MOESM1_ESM.docx]

**Supporting Information**

**Chitosan-based films with cannabis oil as a base material for wound dressing application**

**Dorota Chełminiak-Dudkiewicz^1^*, Aleksander Smolarkiewicz-Wyczachowski^1^, Kinga Mylkie^1^, Magdalena Wujak^2^, Dariusz T. Młynarczyk^3^, Paweł Nowak^1^, Szymon Bocian^4^, Tomasz Gośliński^3^, Marta Ziegler-Borowska^1^***

^1^Department of Biomedical Chemistry and Polymer Science, Medicinal Chemistry Research Group, Faculty of Chemistry Nicolaus Copernicus University in Torun, Gagarina 7, 87-100 Torun, Poland

^2^Department of Medicinal Chemistry, Faculty of Pharmacy, Nicolaus Copernicus University in Torun, Collegium Medicum, Jurasza 2, 85-089 Bydgoszcz, Poland

^3^Chair and Department of Chemical Technology of Drugs, Poznan University of Medical Sciences, Grunwaldzka 6, 60-780 Poznan, Poland

^4^Department of Environmental Chemistry and Bioanalysis, Faculty of Chemistry, Nicolaus Copernicus University in Torun, Gagarina 7, 87-100 Torun, Poland

* Email: [D.CH-D](about:blank).: [dorotachd@umk.pl](mailto:dorotachd@umk.pl), M.Z-B.: martaz@umk.pl

**Methods**

**Biocompatibility evaluation using L929 mouse fibroblasts**

For the extract test, films prepared on 24-well plates were incubated in 1 mL of the growth medium for 24 h at 37 °C in a humidified atmosphere with 5% CO2. L929 fibroblasts were seeded into 96-well plates at a density of 1 × 10^4^/cm^2^. After 24 h cell culture, the medium was replaced with the control growth medium or extracts obtained from biomaterials. The cells were grown for a further 24 or 72 h. Further, L929 fibroblasts were seeded on the films at a density of 1 × 10^4^/cm^2^ and allowed to grow for 24 and 72 h. The effect of the biomaterials extracts on the L929 cell viability was assessed using the MTT assay which is based on the reduction of 3- (4,5-dimethylthiazol- 2-yl)-2,5-diphenyltetrazolium bromide (MTT) by mitochondrial dehydrogenases of viable cells. At the end of the experiment (24 or 72 h treatment), the medium was removed and 100 μL of 0.5 mg/mL MTT (Sigma-Aldrich) prepared in the fresh growth medium was added to each well. After 3 h of incubation at 37 °C in a humidified atmosphere with 5% CO2, the solution was removed, 100 μL of 2-propanol with 0.04N HCl was added to each well to dissolve the produced purple formazan crystals, and the plates were shaken for 15 min at 100 rpm. Then, the absorbance was measured at a wavelength of 570 nm with 288 background subtraction at 690 nm, using a microplate reader (Multiskan Spectrum; Thermo Scientific, Waltham, MA, USA). The number of viable cells was calculated relative to the control cells growing in the growth medium in the absence of the biomaterial’ extract (extract test). The experiments were performed at least in triplicate.

**Figure S1:** Structure of the cannabidiol


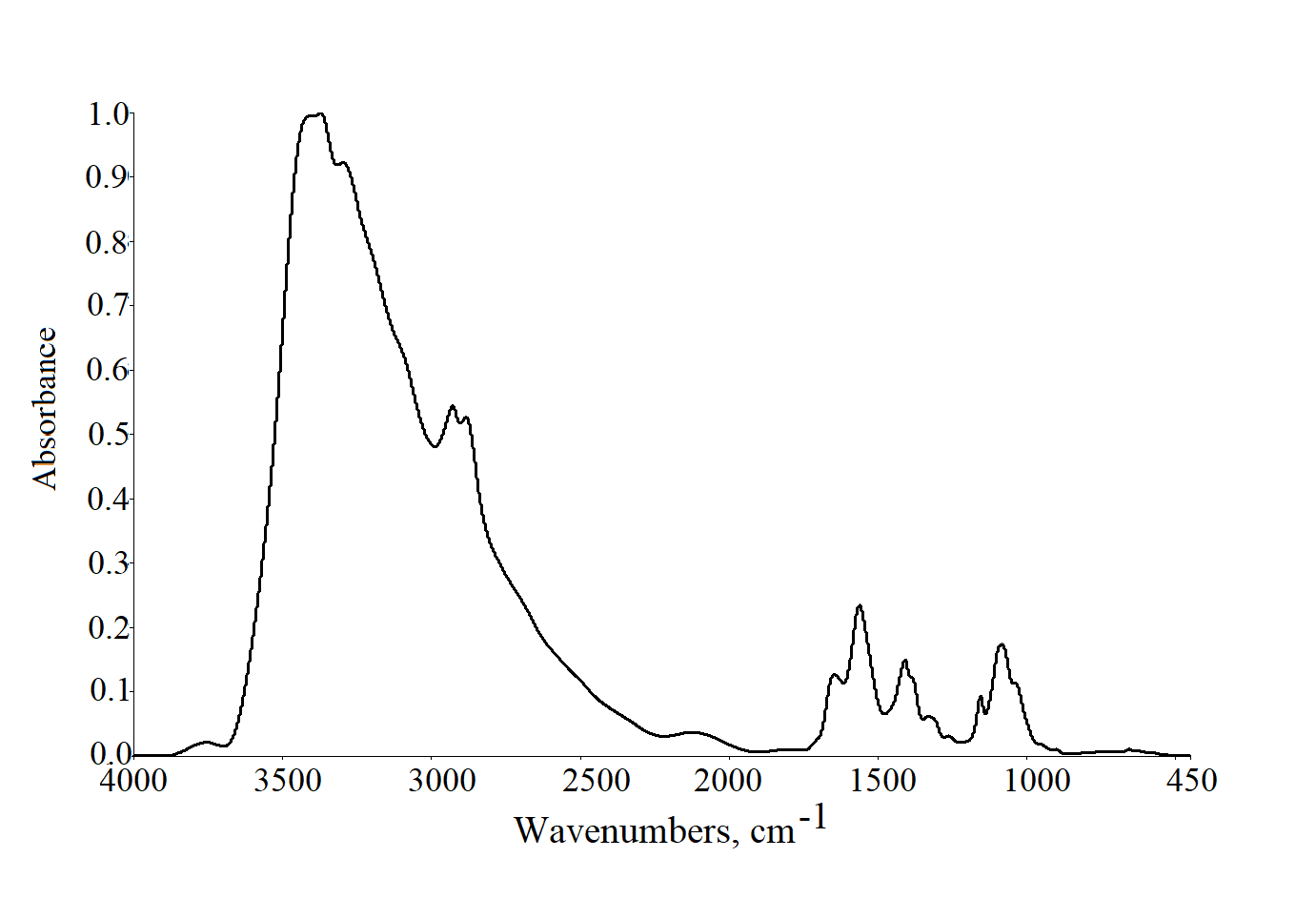


| **Sample** | **First stage** | | **Second stage** | | | **Third stage** | | | | **Residue at 600°C (%)** |
| --- | --- | --- | --- | --- | --- | --- | --- | --- | --- | --- |
|  | *T*_max_  (°C) | Δ*m*  (*%*) | *T*_o_ (°C) | *T*_max_ (°C) | *Δm* (%) | | *T*_o_ (°C) | *T*_max_ (°C) | Δ*m* (*%*) |  |
| **CS** | 69 | 10 | 128 | 188 | 7 | | 222 | 286 | 52 | 31 |
| **1CBD-CS** | 72 | 9 | 130 | 187 | 8 | | 223 | 287 | 53 | 30 |
| **5CBD-CS** | 76, 86 | 9 | 138 | 189 | 7 | | 210 | 280 | 57 | 27 |
| **10CBD-CS** | 64, 86 | 8 | 139 | 191 | 7 | | 210 | 280 | 59 | 26 |

**Figure S2:** FT-IR spectrum of chitosan film

**Table S1.** Thermal parameters of the pure chitosan and the obtained materials


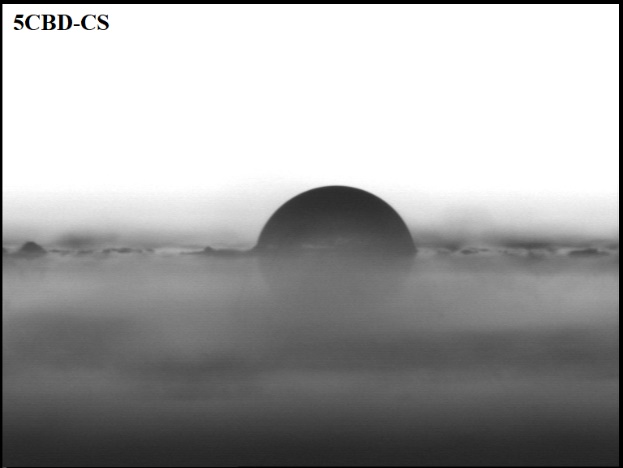

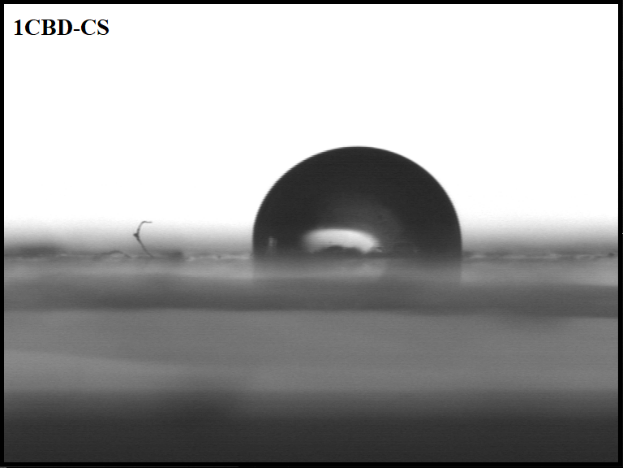


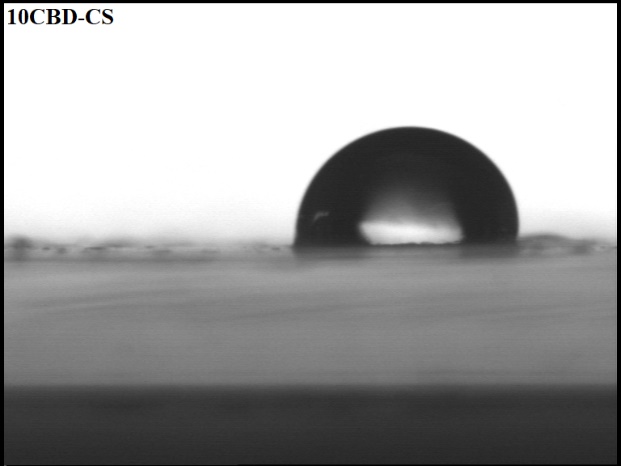


**Figure S3:** The results of the contact angle with water measurements of the obtained films.

**Table S2.** Surface characterization of the obtained materials. a, b, c indicate p < 0.05 when compared to the corresponding CS, 1CBD-CS and 5CBD-CS, respectively.

| **Sample** | **Average Contact Angle**  **[θ, °]** | | **Surface Free Energy [mJ/m^2^]** | | |
| --- | --- | --- | --- | --- | --- |
|  | Measuring Liquid | |  |  |  |
|  | Glycerin | Diiodomethane | γ_s_ | γ_s_^d^ | γ_s_ ^p^ |
| CS | 81.9±0.03 | 55.7±0.04 | 30.70±0.29 | 27.46±0.26 | 3.23±0.04 |
| 1CBD-CS | 60.6±0.03^a^ | 20.5±0.07^a^ | 48.20±0.16^a^ | 41.03±0.03^a^ | 7.17±0.01^a^ |
| 5CBD-CS | 62.7±0.06^a,b^ | 13.6±0.03^a,b^ | 49.26±0.39^a^ | 43.61±0.20^a,b^ | 5.66±0.13^a,b^ |
| 10CBD-CS | 59.9±0.03^a,b,c^ | 22.7±0.03^a,b,c^ | 47.81±0.26^a,c^ | 40.06±0.53^a,c^ | 7.75±0.03^a,b,c^ |


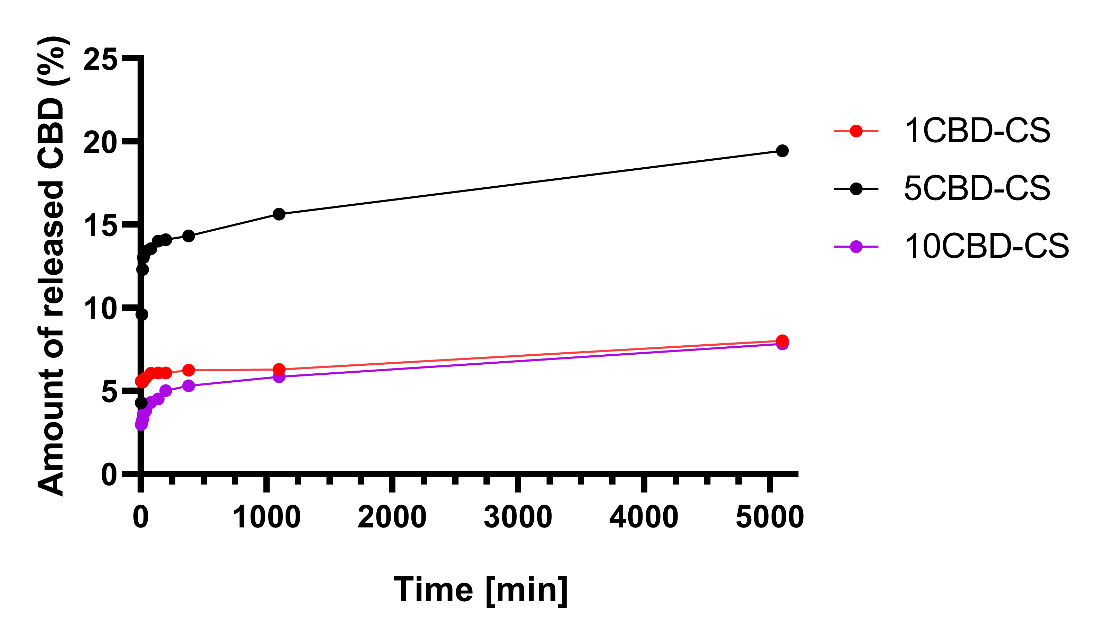


**Figure S4:** Release of the CBD from the obtained materials

**Table S3.** Amount of HSA bound on the surface of the obtained films

| Incubation time  [min] | Amount of bounded HSA [mg/cm^2^] | | | |
| --- | --- | --- | --- | --- |
|  | **Sample** | | | |
|  | **CS** | **1CBD-CS** | **5CBD-CS** | **10CBD-CS** |
| 1 | 0.035 | 0.059 | 0.088 | 0.117 |
| 2 | 0.041 | 0.088 | 0.159 | 0.178 |
| 3 | 0.045 | 0.105 | 0.208 | 0.212 |
| 4 | 0.048 | 0.117 | 0.223 | 0.237 |
| 5 | 0.048 | 0.129 | 0.237 | 0.254 |
| 10 | 0.053 | 0.143 | 0.250 | 0.280 |
| 15 | 0.055 | 0.150 | 0.255 | 0.286 |
| 30 | 0.057 | 0.162 | 0.258 | 0.293 |
| 60 | 0.061 | 0.167 | 0.260 | 0.300 |
| 120 | 0.063 | 0.168 | 0.265 | 0.303 |
| 1440 | 0.072 | 0.192 | 0.269 | 0.305 |


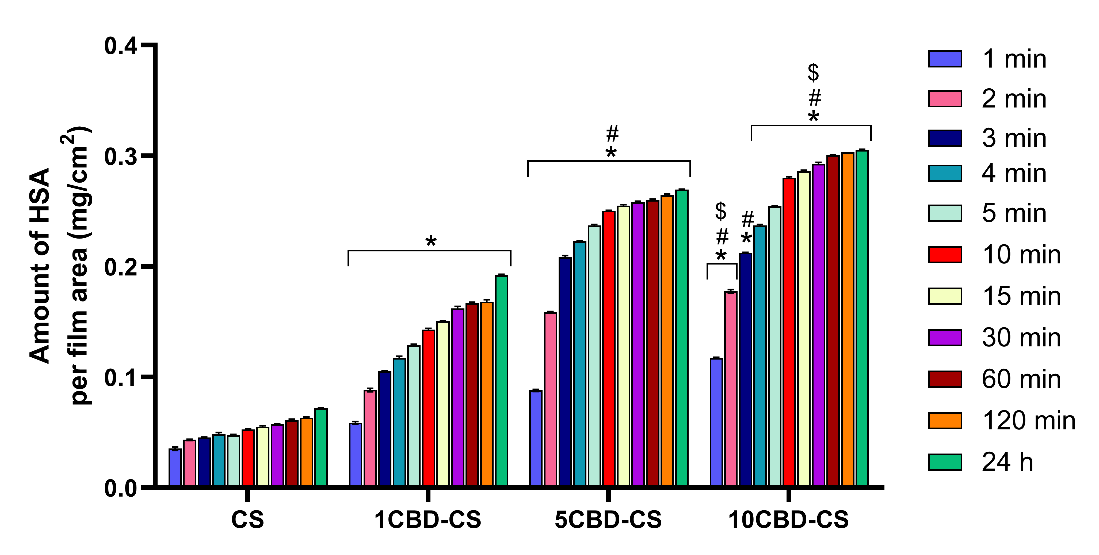


**Figure S5:** Amount of bounded human serum albumin at the surface of obtained films. ^*, #, $^ indicate *p* < 0.05 when compared to the corresponding CS, 1CBD-CS and 5CBD-CS, respectively.

**Table S4**: Amount of released of CBD from the obtained materials

| **Time [min]** | **Release of CBD [%]** | | |
| --- | --- | --- | --- |
|  | **1CBD-CS** | **5CBD-CS** | **10CBD-CS** |
| **5** | 5.57 | 4.28 | 2.96 |
| **10** | 5.59 | 9.60 | 3.05 |
| **15** | 5.53 | 12.30 | 3.26 |
| **20** | 5.67 | 13.00 | 3.57 |
| **40** | 5.79 | 13.39 | 3.80 |
| **80** | 6.05 | 13.55 | 4.30 |
| **140** | 6.07 | 14.02 | 4.51 |
| **200** | 6.07 | 14.10 | 5.02 |
| **380** | 6.24 | 14.32 | 5.30 |
| **1100** | 6.28 | 15.64 | 5.84 |
| **5100** | 8.01 | 19.44 | 7.02 |


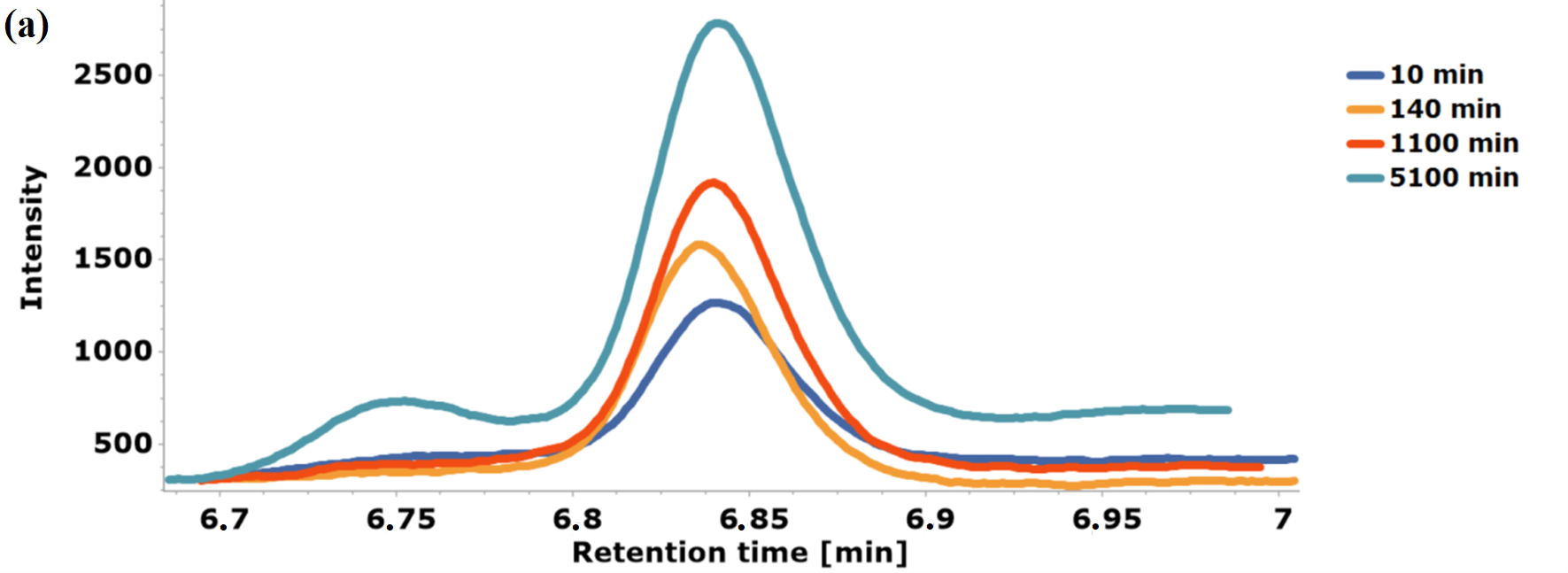


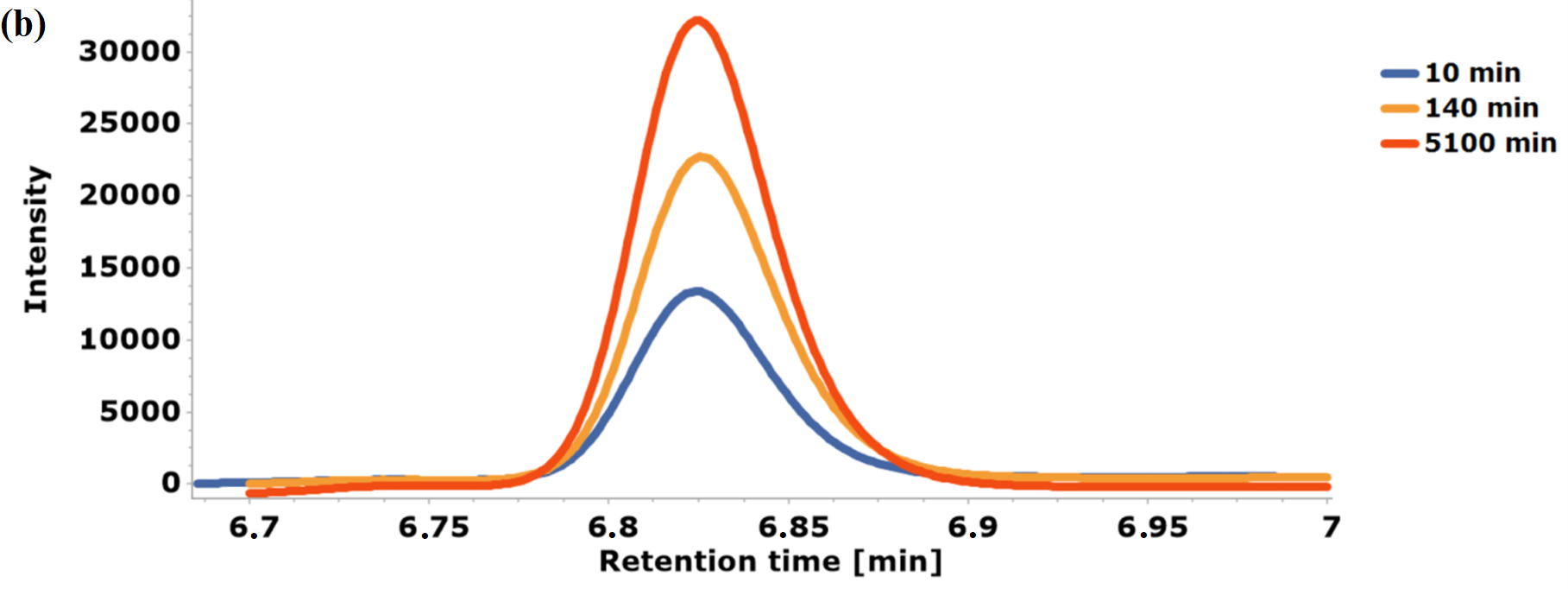


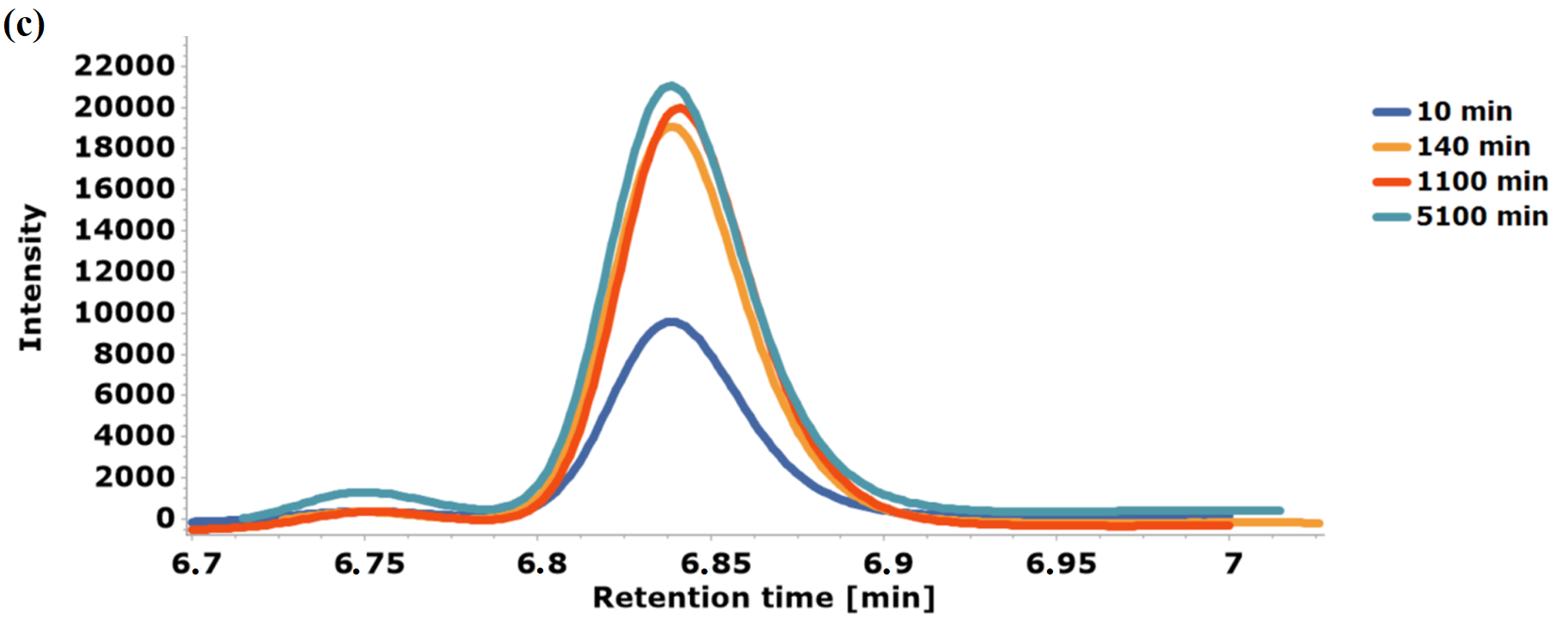


**Figure S6:** HPLC chromatogram of **(a)** 1CBD-CS, **(b)** 5CBD-CS, and **(c)** 10CBD-CS films


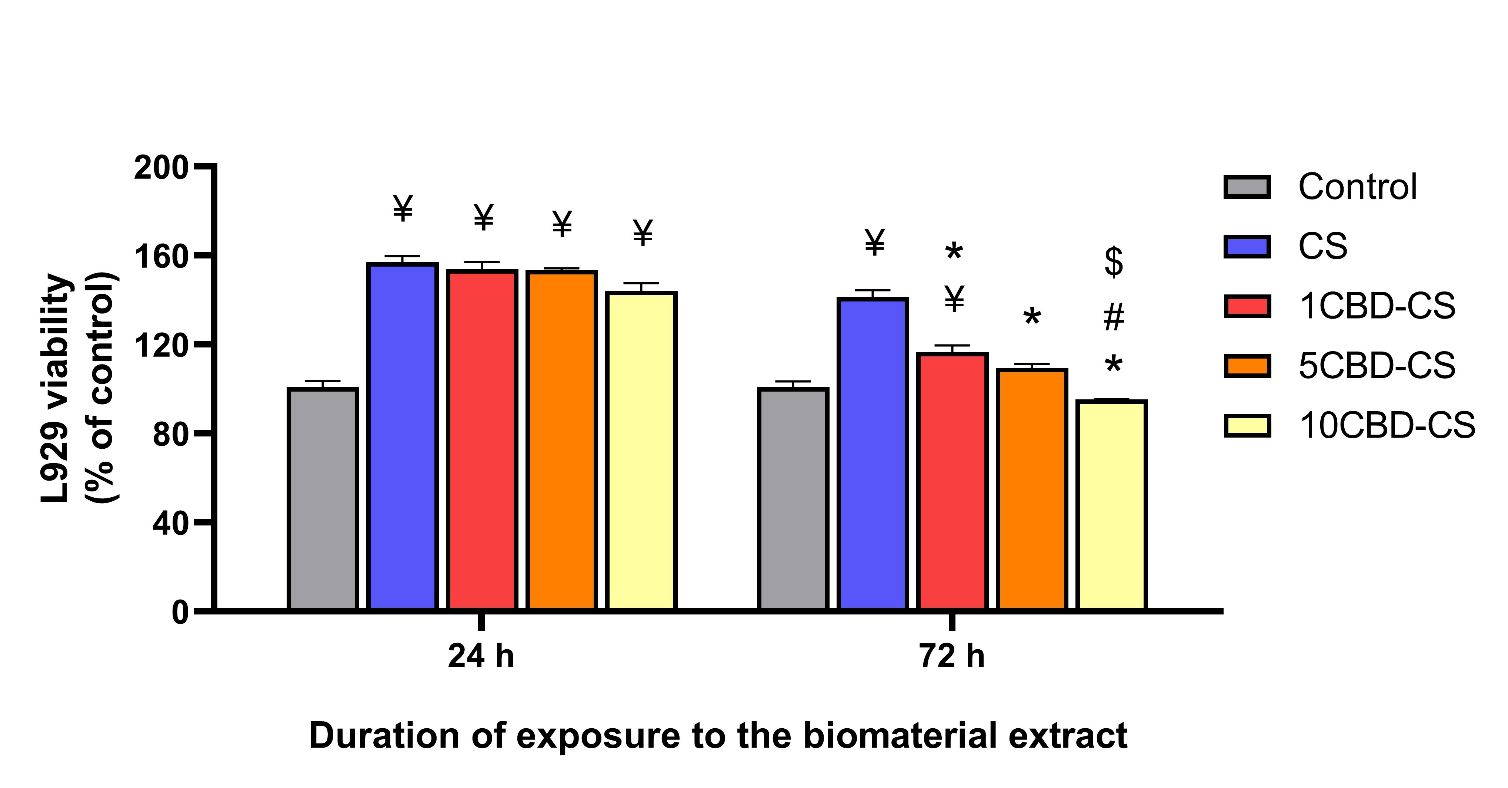


**Figure S7:** Cytotoxicity evaluation in L929 fibroblast cell culture. The viability of L929 cells 697 exposed to the biomaterial extracts for 24 and 72 h (extract test). The results are presented as the 699 percentage of the cell viability compared to the control cells cultured in the growth medium 700 only for the indicated time point. ¥, *, #, $ indicate p < 0.05 when compared to the corresponding Control, CS, 1CBD-CS and 5CBD-CS, respectively.
